# A Simple and Effectual Plug in Nasal Hemorrhage

**Published:** 1875-10

**Authors:** T. H. Jewett


A Simple and Effectual Plug in Nasal Hemorrhage.—*By T. H. Jewett, M.D.*—Many years since my master, the venerable and eminent surgeon, Dr. W. Perry, of Exeter, N. H., taught me at the bedside of a patient nearly moribund, the following simple method of arresting nasal hemorrhage:—

Bellocq's instrument and all its contrivances are not to be compared with this of Dr. Perry's, for ease of application and efficiency. It has never failed in my hands, either in hemorrhage or typhoid fever, in that connected with diseases of the heart, or purpura, or nasal hemorrhages of any other origin. Roll up between the thumb and fingers a lock of cotton into a cylinder or little roll, an inch or an inch and a half in length; tie a strong thread to the middle of the roll; bring the two ends of the roll together, and then, opening the nasal orifice by pressing down with the end of the finger its lower margin, pass the middle or folded part of the roll (where the string is tied) in the nostril; next with the blunt end of a lead pencil or stick, press in the cotton roll slowly, along the floor of the nostril, an inch or more, and rest. If the blood passes down into the throat, you may be sure the bleeding spot is behind the roll, so push in your roll further and the blood will cease to pass behind. Then holding on to the string pass some loose cotton into the nostril, and push it in and along, with the pencil, down to the plug. The cotton will swell with the moisture, compress the bleeding surface, and arrest the hemorrhage. It is well to let the plug remain for two or three days. The string attached to the cotton may be carried up around the alæ nasi, to the side of the cheek, and fastened with a strip of adhesive plaster. In a day or two the mucus or natural secretions of the nasal surfaces will loosen the plug, and it may be easily removed by the string. The dry cotton will, in an ordinary case, answer for the plug. If you choose, you can wet it in liquor ferri persulphate, or cause the plug to be dusted over with pulv. ferri persulphate, or ferric alum, or tannic acid, or any other astringent that may be preferred.—*Phil. Med. and Surg. Reporter.*

